# Risk factors of type-2 diabetes mellitus in rural Wardha: A community based study

**DOI:** 10.4103/0973-3930.44077

**Published:** 2008

**Authors:** Nazli M. Khatib, Zahiruddin S. Quazi, Abhay M. Gaidhane, Trupti S. Waghmare, R. C. Goyal

**Affiliations:** Department of Physiology, J N Medical College, Datta Meghe Institute of Medical Sciences University, Sawangi (M), Wardha MS, India; 1 Department of Community Medicine, J N Medical College, Datta Meghe Institute of Medical Sciences University, Sawangi (M), Wardha MS, India

**Keywords:** Risk factors for diabetes mellitus, rural population, type-2 diabetes mellitus

## Abstract

**CONTEXT:**

The rise in diabetes mellitus (DM) portends a disaster of major proportion worldwide. AIM: To study the determinants of type-2 DM in people who are ≥45 years of age by selective screening methodology in rural area of Wardha district.

**SETTINGS AND DESIGN:**

A cross-sectional study in a rural area of Wardha district.

**METHODOLOGY:**

A cross-sectional population-based study was conducted among those who are at risk of developing DM, as per the WHO guidelines on Laboratory Diagnosis and monitoring of Diabetes Mellitus 2002. Blood glucose estimation was done using a blood glucose meter.

**STATISTICAL ANALYSIS USED:**

Multiple Logistic Regression.

**RESULTS:**

Eight point four nine percent of the 306 persons above the age of 45 years were diabetic. This study also revealed that the proportion of people diagnosed with DM increases with increasing age groups. Mean fasting and post meal blood glucose level (in mg%) among the study participants (nondiabetics) were 83.6 ± 1.6 and 129.9 ± 1.9 and mean fasting and post meal blood glucose level among diabetics were 138.8 ± 2.1 and 220.7 ± 1.9, respectively. The difference between the post meal blood glucose level among the diabetics and nondiabetics was statistically significant. The proportion of diabetics was more among those who had family history of diabetes (8.6%), BMI more than 25 (24.1%) and those with sedentary lifestyle (10.4%).

**CONCLUSIONS:**

Implementation of preventive measures to reduce the burden of diabetes is needed. Identification of the environmental factors adversely related to glucose intolerance helps evolve preventive strategies.

## Introduction

Prevalence of diabetes mellitus (DM) and impaired fasting glucose are reported to be highly variable among rural and urban population in India. A number of epidemiological studies with varying sample sizes have reported prevalence of diabetes at different geographical areas ranging from 1.6–12.4%,[1] type-2 diabetes is a global health problem.[2] According to the recent global estimates of the World Health Organization (WHO), there will be 300 million people with diabetes by the year 2025.[2,3] It is estimated that the developing countries will bear the brunt of diabetes epidemic to the extent of 77% of the global burden, in the 21st century.[4] Number of diabetics in India was 31.7 million in the year 2002[5] and it is estimated that number of diabetics in 2030 will be 79.4 million in India.[6,7]

The prevalence of diabetes in Indian adults was found to be 2.4% in rural and 4–11.6% in urban dwellers.[6] Today, diabetes no longer remains a disease of the high socioeconomic status or confined to urban area. However, not enough prevalence studies have been conducted in India for rural population. American Diabetes Association has proposed the screening of all patients aged over 45 years by measuring fasting blood glucose every three years, in addition to screening patients from high-risk groups and younger patients with hypertension, obesity, a family history of diabetes in a first-degree relative, or a family history of gestational diabetes.[7] Considering the above facts, this study was undertaken to study the distribution of determinants of type-2 diabetes among rural population at risk of developing DM in Deoli Taluka of Wardha District, Maharashtra.

## Methodology

### Study settings

This study was undertaken, in Deoli village, a rural teaching area of Department of Community Medicine, Datta Meghe Institute of Medical Sciences University (NAAC Accredited Grade A) situated 18 km away from Wardha City. The local institutional Ethics Board approved the study protocol.

A cross-sectional population-based study was conducted among those who are at risk of developing DM as per the WHO guidelines on Laboratory Diagnosis and monitoring of Diabetes Mellitus 2002.[8]

### Sample size

Deoli has a population of 15,878 with 3,370 households. The village Deoli was divided into 17 wards and of these, 9 wards (50%) was randomly selected for the study purpose.

### Inclusion criteria

Selective screening methodology was used to detect those who are at risk for diabetes. Criteria for selective screening for diabetes for the individuals as per WHO guidelines included:[9]

- typical symptoms of diabetes,
- first-degree relatives with diabetes,
- overweight (BMI ≥ 25 kg/m^2^),
- women who had delivered baby weighing ≥4.5 kg or had gestational DM,
- hypertensive (≥140/90 mm Hg),
- raised serum triglyceride and cholesterol levels and
- prior history of IGT or IFG

### Exclusion criteria

- known cases of type-2 diabetes,
- individuals who declined for informed consent, and
- not available at home even after repeat second visit.

### Sampling

To select the household, systematic random sampling was done. A list of the households was made in all the selected wards. Thereafter, a random number was chosen by taking the last digit of the currency note and that was selected as the first household, and subsequently, every alternate household was selected. Similar procedure was followed for all the wards. The final sample included 306 individuals, with 171 men and 135 women, after selective screening methodology.

### Data collection

On visiting the selected house, the investigator found out if there are any members aged ≥45 years of age, having any of the above mentioned risk factors. On affirmation, the participant was included in the study after the written informed consent.

Data were collected using a pre-tested structured interview schedule. During the interview, information about sociodemographic status, family history of DM, physical activity, and other known risk factors for diabetes were asked.

After the interview, participants were informed about procedural details of the blood sugar investigation. Participants were motivated to fast overnight and its importance was stressed for correct blood sugar recording. On the next day, the healthcare worker and laboratory technician visited their houses for sample collection and test for the fasting blood glucose by Glucometer. Thereafter, 75 gm powdered glucose in a glass of water was given to participants to estimate the postprandial blood sugar after one hour. The participants were informed about the result of the blood test and necessary advice was given and referral was done to a tertiary health care centre for further management, if needed.

Blood glucose estimation was done using Glucometer (Bayer Corporation-Principle sensor, Calibrated for plasma glucose). Two microliters of the sample was collected by sip-in technique. Test time was 30 sec, test interval g/L- 0.4- 0.5). During the field survey, all the instruments were calibrated each morning using standard solutions and were checked with check-strips after every 20 measurements. All control values were within recommended ranges. The Diabetes Control and Complications Trial (DCCT) clearly demonstrated the benefits of normal or near-normal blood glucose levels by using Glucometer (Bayer Corporation) and the instrument fulfils the defined essential requirements as per standardizing organizations (ISO, CEN).[6,10]

Data were analyzed and presented as simple percentages and proportions. Test of significance was applied wherever necessary. SPSS version 10.05 was used.

### Operational definitions

Hypertension: as per JSM VII criteria.[8]

Obesity: BMI: Normal, 18.5–24.9 kg/m^2^; overweight, 25–29.9 kg/m^2^; obese, ≥30.0 kg/m^2^.

Diabetes: Criteria for the diagnosis of diabetes, proposed by WHO.[9]

## Results

The village Deoli has 3,370 households with a total population of 15,878. Of these, 306 persons of more than 45 years of age and who had at least one or more risk factors for DM, as per WHO guidelines,[9] participated in the study.

Majority belonged to Hindu religion (66%) and the rest belonged to Muslim (11%) and Buddhist (23%) religions, respectively. Twenty six (8.5%), of the 306 participants were diagnosed as diabetics at the time of the survey; mean fasting and post meal blood sugar level (in mg%) of all the study participants were 81.7 (± 3.2) and 118.6 (± 2.5), respectively [Table 1].

**Table 1:** Mean blood sugar level of participants (mean ± SD)

	Number of patients	Mean fasting blood sugar (mg%)	Mean post meal blood sugar (mg%)
Nondiabetics	280	81.7 ± 3.2	118.6 ± 2.5
Diabetics	26	138.8 ± 2.1	220.7 ± 1.9
Total	306	83.6 ± 1.6	129.9 ± 1.9

The mean fasting and post meal blood sugar level (in mg%) among nondiabetics were 81.7 ± 3.2 and 118.6 ± 2.5 and among diabetics were 138.8 ± 2.1 and 220.7 ± 1.9, respectively. The difference in the post meal blood glucase level among diabetics and non-diabetics was found to be significant [Table 1].

The current study also revealed that the proportion of people diagnosed with DM increases with increasing age groups. Out of 51 participants aged >60 years, 11.8% were diabetic as compared to 9.2% and 6.4% in the age group of 51–60 years and 45–50 years, respectively [Table 2].

**Table 2:** Distribution of risk factors among study participants

Risk factors			No. (%)	Diabetics (%)
Age group (years)				
45–50			125 (40.8)	8 (6.4)
51–60			130 (42.5)	12 (9.2)
>60			51 (16.7)	6 (11.8)
Gender				
Male			171 (55.9)	15 (8.8)
Female			135 (44.1)	11 (8.1)
Family history of diabetes				
No history			76 (24.5)	6 (7.9)
Family history			114 (37.3)	10 (8.8)
Not known			116 (37.9)	10 (8.6)
BMI/(Obesity)				
<18.5			156 (51.0)	3 (1.9)
18.5–24.9			92 (30.1)	9 (6.5)
>25			58 (18.9)	14 (24.1)
Blood pressure				
	Systolic	Diastolic		
Normal	<140	< 90	81 (26.5)	7 (8.6)
Grade I	140–159	90–99	92 (30.1)	8 (8.6)
Grade II	160–179	100–109	101 (33.1)	9 (8.9)
Grade III	>180	>110	32 (10.4)	2 (6.2)
Lifestyle				
Sedentary			96 (31.4)	10 (10.4)
Nonsedentary			210 (68.6)	16 (7.6)
Substance abuse				
Nil			21 (6.9)	4 (19.0)
Only Alcohol			51 (16.6)	5 (9.8)
Only smoking			112 (36.6)	6 (5.6)
Both			122 (39.9)	11 (9.0)

Of the 26 diabetics, 15 were men and 11 were women. The gender-wise distribution of diabetes among study subjects was also not statistically significant (P = 0.89). Ten (38.4%) of 26 diabetics gave family history of diabetes and almost similar numbers of diabetics were not aware of their family history. Twenty three percent diabetic participants did not have family history of diabetes.

The study also revealed that as the BMI increases, the proportion of diabetes also increases. Twenty four percent participants with BMI >25 were diabetics as compared to 6.5 and 1.9 among those with BMI of 18.5–24.9 and <18.5, respectively.

Nineteen (8.5%) of the 255 subjects with hypertension of various stages (as per JSM VII criteria) were diabetic and seven (8.6%) of the 81 were normotensive. The association of hypertension and type-2 DM in our study was not statistically significant.

Eleven (9%) of the 122 participants with history of alcoholism and smoking were diabetics as compared to four out of those who did not give history of any addiction.

## Discussion

Of the 306 individuals enrolled in the study, 26 (8.4%) were diabetic, almost similar to the study conducted in Turkey (7.2%).[10] However, the prevalence is lower than that of rural population of Pakistan (11.1%) and Hawaii (20.4%),[11,12] but higher than in Mongolia (2.9%).[13]

In this study, even though more males, as compared to females, were diabetic, the sex distribution among diabetics was not statistically significant (*P* > 0.05). A study conducted in Turkey and Pakistan has found high prevalence of type-2 DM in women.[12,14] Most of the diabetics were in the age group of 50–60 years, this finding was almost similar to the study conducted by Ramachandaran in rural South India, wherein 9.9% out of 588 subjects of above 60 years of age had diabetes.[11]

Most of the studies observed that, regardless of ethnicity, this metabolic disease had increased with economic development related to affluent lifestyle, excess calorie intake and less physical activities resulting from the embrace of a more modernized lifestyle in favor of a traditional lifestyle.[15–18] The preponderance of diabetes in the rural rich indicates the same.

In rural India, developmental changes have influenced the lifestyle of rural people. Therefore, higher prevalence of type-2 DM in study population may be primarily due to environmental factors, apart from the genetic predisposition.[19–20]

The study revealed that family history of diabetes, increasing age and BMI (obesity) are few important risk factors found to be more among diabetics as compared to nondiabetics. For type-2 diabetes, this is not an unusual finding.[11,20,21]
